# Aspectos fundamentales en la solicitud y determinación de la lipoproteína(a) en el laboratorio clínico

**DOI:** 10.1515/almed-2024-0090

**Published:** 2025-03-03

**Authors:** Teresa Arrobas Velilla, Carla Fernández Prendes, Núria Amigó Grau, Pilar Calmarza, Silvia Camós Anguila, Beatriz Candas Estébanez, María José Castro Castro, David Ceacero, Irene González Martínez, María Martín Palencia, José Puzo Foncillas, Carlos Romero Román, en nombre de la Comisión de Lipoproteínas y Enfermedades Cardiovasculares de la SEQCML

**Affiliations:** Hospital Universitario Virgen Macarena, Sevilla, España; Servicio de Análisis y Bioquímica clínica. Laboratori Clínic Metropolitana Nord, 16514 Hospital Universitari Germans Trias i Pujol, Badalona, Barcelona, España; Department of Basic Medical Sciences, Rovira i Virgili University, Reus, España; Center for Biomedical Research in Diabetes and Associated Metabolic Diseases (CIBERDEM), Instituto de Salud Carlos III, Madrid, Comunidad de Madrid, España; Biosfer Teslab, Reus, España; Servicio de Bioquímica Clínica, Hospital Universitario Miguel Servet, Zaragoza, Aragón, España; Centro de Investigación en Red en Enfermedades Cardiovasculares (CIBERCV), Instituto de Investigacion Sanitaria Aragon, Zaragoza, España; Servicio de Análisis Clínicos – Bioquímica – Laboratori Clínic, Hospital Universitari de Girona Doctor Josep Trueta, Girona, Catalunya, España; Laboratorio Clínico, Hospital de Barcelona, Barcelona, Catalunya, España; Facultat de Medicina, UVic-UCC, Vic, España; Facultat de Ciències, UVic-UCC, Vic, España; Core Bioquímica, Laboratori Clínic Territorial Metropolitana Sud, Hospital Universitario de Bellvitge, L’Hospitalet de Llobregat, España; Servicio de Análisis Clínicos, Hospital Universitario 12 de Octubre, Madrid, Comunidad de Madrid, España; Servicio de Análisis Clínicos, Hospital Universitario de Burgos, Burgos, Castilla y León, España; Servicio de Análisis y Bioquímica Clínica. Unidad de Lípidos, Hospital General Universitario San Jorge de Huesca, Huesca, España; Facultad de Ciencias de la Salud y Deporte, Huesca, España; Hospital General Universitario de Albacete, Albacete, Castilla-La Mancha, España

**Keywords:** colesterol, determinación, isoformas, lipoproteína(a), recomendaciones

## Abstract

Las enfermedades cardiovasculares continúan siendo la principal causa de muerte en España, lo que sugiere la necesidad de estudiar la presencia de nuevos factores de riesgo que puedan estar contribuyendo a aumentar el riesgo cardiovascular. La lipoproteína(a) (Lp(a)) se ha asociado con un mayor riesgo de desarrollar estenosis valvular aórtica, insuficiencia cardíaca, ictus isquémico, cardiopatía isquémica y enfermedad arterial periférica. La hiperlipoproteinemia(a) es un problema de salud generalizado. Entre el 10 % y el 30 % de la población mundial presenta valores de Lp(a) superiores a 50 mg/dL. La evidencia científica acumulada en los últimos años ha confirmado la existencia de una asociación independiente entre la concentración de Lp(a) y el riesgo de presentar un evento cardiovascular arteriosclerótico. Este hallazgo, unido al creciente desarrollo de nuevas terapias específicas para reducir la Lp(a), ha incrementado notablemente el interés por su medición. El objetivo de este documento es, en base a la evidencia actual, informar sobre a qué pacientes se debería medir la Lp(a), cuáles son los métodos de medición recomendados, las concentraciones deseables y la utilidad de su medición en la reclasificación de pacientes según su riesgo cardiovascular.

## Introducción

Las enfermedades cardiovasculares y del sistema circulatorio continúan siendo la principal causa de muerte en España [1]. Además, en un porcentaje significativo de la población se desarrollan eventos cardiovasculares precoces o recurrentes, a pesar de realizar seguimiento de los factores de riesgo (FR) clásicos como la hipertensión arterial, la diabetes mellitus (DM), o tabaquismo, la disponibilidad de tratamientos farmacológicos óptimos dirigidos a reducir la concentración de colesterol de las lipoproteínas de baja densidad (LDL-C) y un estrecho seguimiento asistencial [2], 3].

Esto sugiere la necesidad de estudiar la presencia de otros FR no tradicionales o independientes que contribuyen a potenciar el riesgo cardiovascular (RCV) o conferir, en pacientes con un adecuado control de los FR clásicos, un riesgo residual para el desarrollo de un evento cardiovascular aterosclerótico [3].

La lipoproteína(a) (Lp(a)) es un FR crucial implicado en el desarrollo de la placa aterosclerótica. El incremento de su concentración se ha asociado con un mayor riesgo de desarrollar estenosis valvular aórtica, insuficiencia cardíaca, ictus isquémico, cardiopatía isquémica y enfermedad arterial periférica [2]. Confiere propiedades proinflamatorias, proateroscleróticas, y procalcificantes que pueden estar relacionadas en parte por los fosfolípidos oxidados (OxPL), transportados preferentemente por la Lp(a) en el plasma [4].

La hiperlipoproteinemia(a) es un problema de salud generalizado en la población. El 10–30 % de la población mundial presenta valores de Lp(a) >50 mg/dL, de los cuales 148 millones se ubican en Europa [5]. Datos obtenidos del estudio Spanish Familial Hypercholesterolaemia Cohort Study (SAFEHEART) muestran que en España aproximadamente un 30 % de los pacientes con hipercolesterolemia familiar (HF) presentaban Lp(a) >50 mg/dL (125 nmol/L) [6]. Estos datos son similares a los obtenidos en Extremadura y Andalucía con un 29.58 % de los pacientes analizados con concentraciones de Lp(a) >50 mg/dL (125 nmol/L) y un 1.52 %, >180 mg/dL. (430 nmol/L) [7].

La Lp(a) fue descrita por primera vez por el médico noruego Kåre Berg en el año 1963 durante un experimento de inmunización de conejos con lipoproteínas de baja densidad (LDL) considerándola como una variante antigénica del LDL-C [8]. No fue hasta una década después, en el año 1970 cuando se identificó una nueva variante electroforética de lipoproteína inicialmente llamada Lp prebeta-1, por la posición que migraba en gel de agarosa, que posteriormente fue identificada como Lp(a) [9], 10]. Se comenzó a poner de manifiesto la mayor implicación de la Lp(a) como FR genético en la enfermedad coronaria [11]. Actualmente está considerada como uno de los FR heredable más importante en la enfermedad cardiovascular [12].

**Figura 1:**
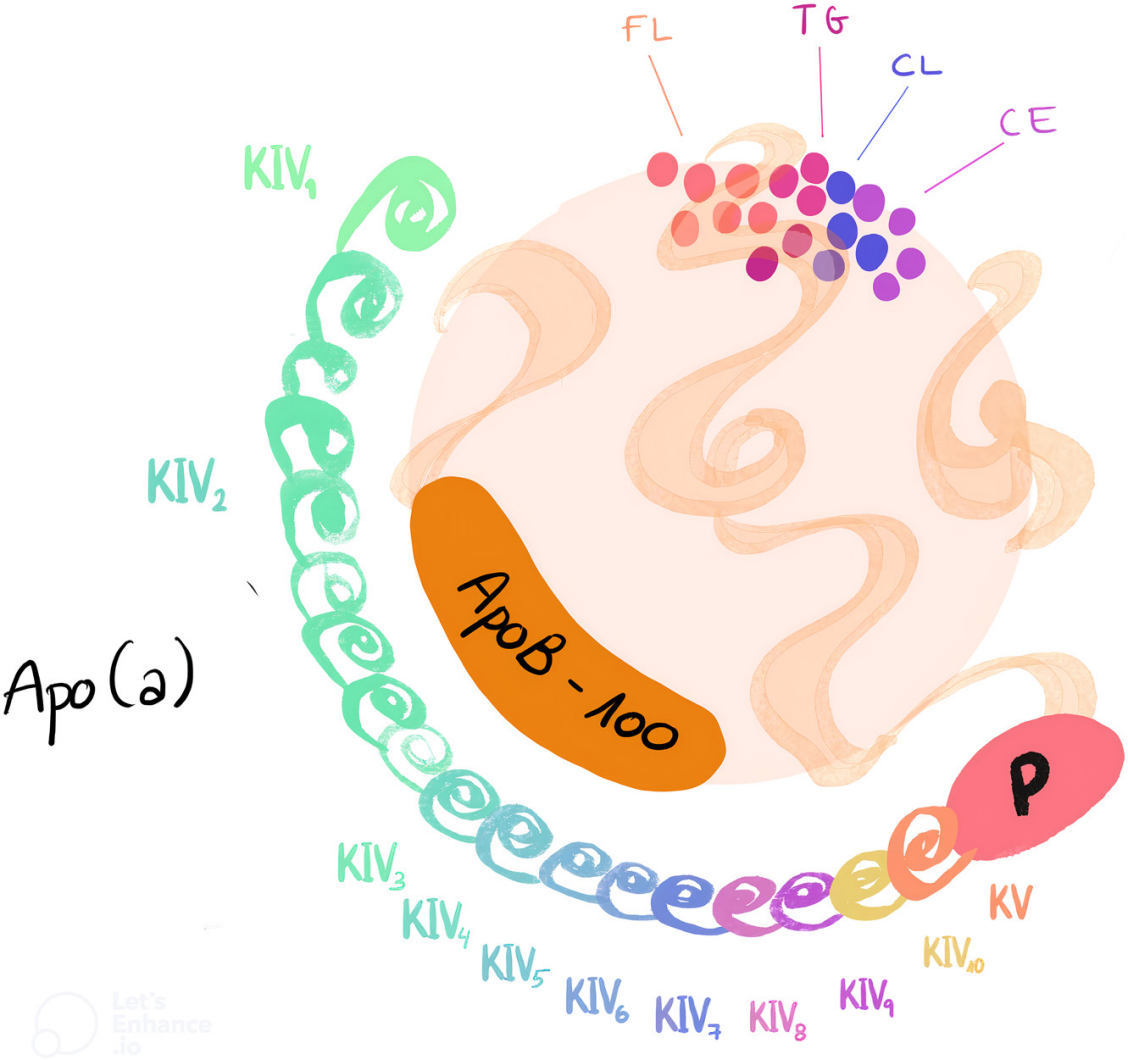
Estructura de una molécula de lipoproteína(a).

La Lp(a) es una lipoproteína plasmática con estructura similar a las LDL en cuanto a su tamaño, composición lipídica y a la presencia de apolipoproteína B100 (apo B100). Además, contiene otra proteína altamente glicosilada y polimórfica denominada apolipoproteína(a) (apo(a)) que se une covalentemente a la apo B100 en proporción equimolar 1:1 [10]. Es sintetizada y secretada por el hígado (Figura 1) [13], 14].

El gen que codifica la Lp(a) se ubica en las posiciones 26 y 27 del brazo largo del cromosoma 6 (6q26-27), se caracteriza por un alto grado de polimorfismo y consta de una repetición exónica variable con un dominio proteico denominado “kringle” (k) [15], que evolucionó a partir del gen del plasminógeno (*PLG*). El plasminógeno presenta una estructura de cinco bucles llamados kringles (KI, KII, KIII, KIV, KV) y un dominio proteasa. A diferencia del plasminógeno, la apo(a) carece de KI, KII y KIII y presenta sólo una copia de KV, un dominio tipo serina proteasa inactivo y diez subtipos de KIV (KIV1 a KIV10) [16] con predominio del subtipo KIV2 repetido en múltiples copias, que son las responsables de la heterogeneidad de tamaño de las isoformas de apo(a), y constituyen la principal causa de su dificultad metodológica [17].

El peso molecular de la apo(a) varía entre 275 y 800 kDa, lo que se atribuye a la existencia de más de 40 isoformas diferentes. Esta variabilidad se debe a que las isoformas de apo(a) se determinan por el número de repeticiones de la secuencia KIV2 en el gen que codifica esta proteína, con un rango que va de 3 a más de 40 repeticiones [13]. Pocos individuos tienen dos alelos de idéntico número de copias en sus genomas. Hasta el 80 % de los pacientes presentan dos isoformas de apo(a) diferentes [17].

La elevada variabilidad biológica interindividual de las concentraciones de Lp(a) se debe al polimorfismo de variación del número de copias (CNV) existente en el locus del gen LPA, lo que explica el 70–90 % de las diferencias observadas en la concentración entre individuos [18], 19].

Además, la concentración de Lp(a) está inversamente relacionada con el tamaño de la isoforma de apo(a), cuanto mayor es el número de copias de KIV2, mayor es el tamaño de la isoforma de apo(a) y menor es la concentración de Lp(a) en plasma [19]. Las altas concentraciones de la isoforma Lp(a) más pequeña, con pocas copias de KIV2, están fuertemente relacionadas con un mayor riesgo cardiovascular [20].

Es aceptado que la concentración de Lp(a) está determinada por su producción y secreción y no por su catabolismo, pero existen aún muchas dudas sobre el lugar de ensamblaje y eliminación [21]. El genotipo de apo(a) determina tanto la tasa de síntesis como el tamaño de apo(a), lo que supone aproximadamente el 90 % de la concentración plasmática. Aunque no se conoce con exactitud dónde ocurre el ensamblaje de esta lipoproteína, sus componentes se sintetizan mayoritariamente en los hepatocitos [17], 21]. Las vías de eliminación de la Lp(a) tampoco están claras, habiéndose sugerido un mecanismo de eliminación en dos pasos, en el que la Lp(a) se desprende de apo(a) en la circulación, aunque no se conocen las reacciones implicadas en este proceso. Además, se postula que, aunque de forma minoritaria, la Lp(a) puede eliminarse a través del receptor de LDL y receptores *scavenger* [18], 21].

Otros factores genéticos que pueden condicionar su concentración son los polimorfismos de un solo nucleótido (SNP), siendo rs3798220 y rs10455872 los más conocidos, y los polimorfismos de repetición de pentanucleótidos [15]. Las concentraciones de Lp(a) varían significativamente entre distintas razas y grupos étnicos, con niveles especialmente elevados en personas de raza negra. Esta variabilidad es más marcada que la observada en otros biomarcadores cardiovasculares [22].

Los factores ambientales tienen poco efecto en la variación de la concentración plasmática de la Lp(a); sin embargo, hay que tener en cuenta algunas causas secundarias que pueden influir en su concentración [23]. En la enfermedad renal crónica (ERC), en la proteinuria en rango nefrótico y en el hipotiroidismo manifiesto, pueden encontrarse concentraciones elevadas de Lp(a). Sin embargo, los trastornos hepáticos suelen dar lugar a concentraciones disminuidas [24]. Además, mientras que los hombres mantienen concentraciones estables de Lp(a) a lo largo de su vida [7], en las mujeres postmenopáusicas se observa un aumento de concentración de Lp(a) respecto a su concentración antes de la menopausia, la cual puede normalizarse con terapias hormonales sustitutivas [19], 23].

## ¿A quién debemos medir la Lp(a)?

Las concentraciones plasmáticas de la Lp(a) permanecen relativamente estables a lo largo de toda la vida de un individuo, debido a su predeterminación genética y se ven poco afectadas por el estilo de vida pudiendo ser un 5–10 % superior en mujeres que en varones [19].

La mayoría de las guías clínicas no recomiendan el cribado universal [24], [25], [26], [27], [28], [29], ya que pese a la fuerte asociación entre las concentraciones elevadas de Lp(a) y ECVA y EVA, actualmente no existe evidencia clínica suficiente para demostrar asociación directa entre la reducción de Lp(a) y la reducción de eventos cardiovasculares, independientemente del LDL-C. Esta situación también se debe a la falta de estandarización de los métodos utilizados en su determinación. En la Tabla 1 se muestran las recomendaciones para el cribado de Lp(a) según las distintas sociedades científicas [25], [26], [27], [28], [29], [30], [31], [32], [33].

**Tabla 1:** Recomendaciones para el cribado de Lp(a) según las diversas sociedades científicas.

Guías de práctica clínica	Recomendaciones de cribado aplicables en la actualidad
2019 ACC/AHA [27]	– Historia familiar de ECVA prematura que no se explica por FR mayores.
2021 NLA [28]	En adultos se recomienda cuantificar la Lp(a) como parte de la evaluación inicial del paciente en los siguientes casos: – Historia personal de ECVA prematura – Antecedentes familiares de ECVA prematura o Lp(a) elevada – LDL-C >190 mg/dL – Sospecha de HF – Riesgo muy alto de ECVA – Pacientes en tratamiento lipídico, con dosis máxima de estatinas±ezetimiba, con niveles de LDL-C >70 mg/dL y que podrían beneficiarse de la terapia con inhibidores de PCSK9. – En jóvenes (<20 años): – Con sospecha clínica de HF o genéticamente confirmada. – Individuos con familiares de primer grado con enfermedad cardiovascular (ECV) – Causa desconocida de ictus isquémico – Antecedentes familiares de primer grado con Lp(a) elevada
2019 HEART UK [25]	– Historial personal o familiar de ECVA prematura (<60 años) – Familiar de primer grado con niveles elevados de Lp(a) (>200 nmol/L) – Hipercolesterolemia familiar (HF) u otra dislipemia genética – Estenosis calcificada de la válvula aórtica – Riesgo intermedio o límite de eventos de ECVA a 10 años, para la reclasificación del riesgo
2021 NSFA [29]	La cuantificación de Lp(a) está recomendada en los siguientes casos: – Pacientes con alto RCV o antecedentes familiares de cardiopatía coronaria prematura – Diagnóstico de HF – Sospecha de dislipemia aterogénica, diabetes tipo 1, diabetes tipo 2 o ERC No se recomienda medir Lp(a) en presencia de insuficiencia hepática, inflamación o enfermedad intercurrente. La determinación no debe repetirse si ya se realizó en condiciones basales adecuadas
2019 ESC/EAS [30]	– Se debe considerar la cuantificación de Lp(a) al menos una vez en la vida de una persona adulta para identificar a los individuos con una concentración de Lp(a) heredada >180 mg/dL (>430 nmol/L) que puede tener un riesgo vitalicio de ASCVD, equivalente al riesgo de hipercolesterolemia familiar heterocigótica (HFe). – Se debe considerar la determinación de Lp(a) en pacientes seleccionados con historia familiar de ECV prematura y para la reclasificación de personas que están en el límite entre riesgo moderado – alto.
2021 Sociedad Cardiovascular Canadiense [31]	– Medir la Lp(a) al menos una vez en la vida – Individuos dentro de la categoría de riesgo intermedio de Framingham (10–19 %) – Antecedentes familiares de ECV prematura
2023 Documento de consenso para la determinación e informe del perfil lipídico en laboratorios clínicos españoles [32]	Se recomienda medir la Lp(a) al menos una vez en la vida. Esta determinación es especialmente relevante en pacientes con ECV prematura, hipercolesterolemia familiar, pobre respuesta al tratamiento con estatinas, estenosis aórtica o eventos isquémicos recurrentes y obviamente en los familiares de pacientes con Lp(a) elevada
2022 Sociedad Europea de Arteriosclerosis [26]	La Lp(a) debe medirse al menos una vez en adultos, preferiblemente en el primer perfil lipídico, para identificar a aquellos con alto riesgo cardiovascular. Esto nos permitiría identificar individuos con concentraciones hereditarias muy altas de Lp(a) >180 mg/dL (>430 nmol/L) que pueden tener un riesgo de por vida de ECVA equivalente al riesgo de Hipercolesterolemia familiar heterocigótica (HFHe)
2024 Consenso sobre Lipoproteina(a) de la Sociedad Española de Arteriosclerosis [33]	Se recomienda la primera determinación de lipoproteína (a) en los siguientes casos: – Pacientes con una manifestación clínica de EVA (pacientes en prevención secundaria) – EVA de cualquier territorio, en especial en casos de presentación precoz – Estenosis aórtica (calcificada) En sujetos <65 años – Hipercolesterolemia familiar (confirmada o sospecha clínica) independiente del resultado de estudio genético para HF – Familiares de primer grado con Lp(a) elevada, Ineludiblemente si caso índice presenta Lp(a) >200 nmol/L, o si el caso índice presenta >100 nmol/L y el paciente presenta otros factores de riesgo CV – Historia familiar de ECV precoz de causa desconocida (familiares de primer grado) – Pobre respuesta al tratamiento con estatinas. Reducción <20 % del c-LDL con estatinas de intensidad media o elevada – Primera valoración de riesgo CV Para mejorar la estratificación del riesgo – Como recomendación general, es aconsejable hacer una determinación de Lp(a) a toda la población al menos una vez en la vida, haciéndola coincidir con otra extracción para un perfil lipídico

ACC/AHA, American College of Cardiology/American Heart Association; NLA, National Lipid Association; HEART UK, HEART UK, The Cholesterol Charity; NSFA, New French Atherosclerosis Society; ESC/EAS, European Society of Cardiology/European Atherosclerosis Society.

La mayoría de las directrices y declaraciones de consenso sugieren una medición al menos una vez en la vida, basada en la consideración de que concentraciones elevadas de Lp(a) son un FR, y coinciden en realizar la determinación de la Lp(a) si existen antecedentes familiares de ECV prematura [15], [24], [25], [26], [27], [28], [29], [30], [31].

La incorporación de la Lp(a) en la evaluación del riesgo global también puede mejorar la estratificación del riesgo. Los resultados deben interpretarse teniendo en cuenta otros FR, para no subestimar el riesgo global absoluto de un evento cardiovascular. La estrategia puede permitir identificar a las personas con elevaciones de Lp(a) menos extremas que pueden presentar un mayor riesgo de ECVA, el cual no se refleja adecuadamente en el Sistema SCORE, o con otras mediciones de lípidos o lipoproteínas. Por lo tanto, debe considerarse en pacientes cuyo riesgo estimado a 10 años de ECVA esté cerca del umbral de riesgo entre moderado y alto [34]^.^

## ¿Hay que medir la Lp(a) en niños y adolescentes?

Las variaciones genéticas que determinan los niveles plasmáticos altos de Lp(a) están presentes desde el nacimiento. Se ha demostrado en varios estudios que la concentración de Lp(a) es baja al nacer, alcanzando valores de adulto en los dos primeros años de vida [30]. La detección temprana de concentraciones elevadas de Lp(a) puede ser clínicamente relevante para recomendar un estilo de vida saludable, que pueda minimizar el desarrollo de la aterogénesis, proceso que comienza ya en la infancia [34], 35].

Las recomendaciones para niños y jóvenes son limitadas y solo respaldan la realización selectiva de la prueba de Lp(a) mediante estudios en cascada familiar [36], 37]. En otras recomendaciones se indica que es necesario medir la Lp(a) en varias ocasiones, ya que sus niveles pueden aumentar hasta la edad adulta [38]. El Panel de Expertos sobre Directrices Integradas para la Salud Cardiovascular y la Reducción del Riesgo en Niños y Adolescentes [39] recomienda medir la Lp(a) en niños ≥2 años con antecedentes familiares de accidente cerebrovascular isquémico o hemorrágico y antecedentes familiares de ECV no explicados por FR clásicos.

La ESC (European Society of Cardiology) en su declaración de consenso [30] recomienda el cribado de Lp(a) en jóvenes cuando hay antecedentes de accidente cerebrovascular isquémico o antecedentes familiares de ECVA prematura o Lp(a) alta y sin otros FR identificables.

La NLA recomienda [28] la detección selectiva de Lp(a) en jóvenes <20 años con HF, ya que pueden estar en riesgo particular de ECVA acelerada, especialmente cuando el LDL-C elevado se acompaña de una Lp(a) elevada, antecedentes familiares de ECV prematura en parientes de primer grado, causas desconocidas de accidente cerebrovascular isquémico y si se encuentran niveles elevados de Lp(a) en padres o hermanos. En algunos artículos se indica la necesidad de la detección en cascada cuando existen antecedentes familiares de Lp(a) muy elevada, antecedentes personales o familiares de ECVA, y HF [19]. La NLA recomienda el cribado en cascada reversa si se encuentran niveles elevados de Lp(a) en niños [28].

Datos limitados sugieren una asociación entre la Lp(a) y la incidencia de accidente cerebrovascular isquémico arterial y tromboembolismo venoso/trombosis venosa en niños, con un riesgo que se duplica con niveles de Lp(a) >30 mg/dL [26].

## ¿Cuándo hay que repetir su determinación?

Las concentraciones plasmáticas de Lp(a) están determinadas principalmente por factores genéticos y se consideran estables a lo largo de la vida. Sin embargo, actualmente se dispone de una cantidad limitada de estudios longitudinales que analicen posibles modificaciones de la concentración de Lp(a) a lo largo del tiempo o establezcan el momento óptimo para su determinación.

Recientemente, se han publicado dos estudios que evidencian cambios en las categorías de riesgo de pacientes tras reanalizar sus valores de Lp(a), considerando factores como el sexo. En estos estudios, los hombres presentaron una variabilidad ligeramente mayor que las mujeres. Además, se identificó la menopausia como un factor condicionante de dicha variabilidad [40] y se destacó la dependencia de la concentración basal como otro elemento relevante [41].

Por lo tanto, no está claro si una medición única de Lp(a) a lo largo de la vida es suficiente para evaluar el riesgo cardiovascular en todos los adultos. Se debería reconsiderar la necesidad de repetir la medición, especialmente en aquellos pacientes cuya concentración de Lp(a) se encuentra en la “zona gris” [41]. Asimismo, se debería indicar una repetición de la prueba en casos de sospecha de causas secundarias que puedan modificar las concentraciones de Lp(a), como procesos agudos con inflamación, enfermedad renal crónica (ERC), síndrome nefrótico, enfermedad hepática crónica, hipotiroidismo, diabetes mellitus (DM) y estado posmenopáusico, o para evaluar la respuesta a intervenciones terapéuticas dirigidas a disminuir sus concentraciones plasmáticas.

En la Tabla 2 se presentan los factores no genéticos que pueden influir en las concentraciones de Lp(a) [42].

**Tabla 2:** Factores no genéticos que pueden influir en la concentración de Lp(a).

Intervenciones y condiciones		Asociación con niveles de Lp(a)
1	**Dieta**	
	a. Reemplazo de grasas saturadas en la dieta con carbohidratos o grasas insaturadas	∼8–20 % de aumento
	b. Dieta baja en carbohidratos y alta en grasas saturadas	∼15 % de disminución
	c. Consumo de alcohol	Sin asociación o disminución menor
	d. Ayuno	Asociación nula
2	**Actividad física** **y ejercicio**	Asociación nula o mínima
3	**Sexo, hormonas** y **condiciones asociadas**	
	a. Sexo	Sin asociación o niveles más altos en mujeres
	b. Hormonas sexuales (endógenas)	Ninguna asociación o asociación menor
	c. Terapia de reemplazo hormonal posmenopáusica	∼20–25 % de disminución
	d. Hipertiroidismo	Disminución de Lp(a); el tratamiento del hipertiroidismo aumenta la Lp(a) en un 20–25 %
	e. Hipotiroidismo	Lp(a) elevada; el tratamiento del hipotiroidismo disminuye la Lp(a) en un 5–20 %
	f. Terapia de reemplazo de hormona de crecimiento	∼25–100 % de aumento
	g. Embarazo	Aumento (hasta 100 %)
	h. Menopausia	Aumento
	i. Hormonas de crecimiento	Aumento (hasta 100 %)
4	**Enfermedad renal crónica**	
	a. Enfermedad renal crónica y hemodiálisis	Lp(a) elevada; nivel 2–4 veces en pacientes portadores de isoformas grandes
	b. Diálisis peritoneal ambulatoria continua	∼2 veces elevado frente a controles
	c. Síndrome nefrótico	∼Aumento de 3 a 5 veces en comparación con los controles
	d. Trasplante de riñón	Reducción significativa; casi normalización
5	**Enfermedad hepática**	
	a. Daño hepatocelular	Disminuido en paralelo con la progresión de la enfermedad
	b. Enfermedad del hígado graso no alcohólico	Asociación inconsistente entre grupos de población
6	**Inflamación**	
	Inflamación	Aumento

## ¿Cuándo está elevada la Lp(a)?

La medición de las concentraciones de Lp(a) mediante un ensayo estandarizado es el método de elección para estimar el riesgo aterogénico asociado a esta lipoproteína. El uso de los SNP para estimar el riesgo no aporta valor añadido a la medición de la proteína biológicamente activa [25], 26].

No existe consenso para establecer un punto de corte de riesgo universal [40], ya que existen diferencias entre los métodos utilizados para su determinación y las unidades de medida. También se plantea la necesidad de establecer rangos específicos en función de la etnia y comorbilidades (enfermedad hepática, ERC, DM). En la Tabla 3 se muestran los puntos de corte recomendados por diferentes sociedades científicas [25], [26], [27], [28], [29], [30], [31], [32].

**Tabla 3:** Puntos de corte recomendados por diversas sociedades científicas que indican riesgo cardiovascular debido a la elevación de Lp(a).

Guías de práctica clínica	Puntos de corte por encima de los cuales existe riesgo cardiovascular por elevación de Lp(a)
2018 ACC/AHA	>125 nmol/L (50 mg/dL)
2019 NLA	>100 nmol/L (40 mg/dL)
2019 ESC/EAS	>430 nmol/L (>180 mg/dL) umbral de riesgo equivalente a HFHe
2019 HEART UK	Riesgo menor: 32–90 nmol/L Riesgo moderado: 90–200 nmol/L Riesgo alto: 200–400 nmol/L Riesgo muy alto: >400 nmol/L
2021 Sociedad Cardiovascular Canadiense	>125 nmol/L (50 mg/dL)

ACC/AHA, American College of Cardiology/American Heart Association; NLA, National Lipid Association; ESC/EAS, European Society of Cardiology/European Atherosclerosis Society; HEART UK, HEART UK, The Cholesterol Charity.

## Métodos de medida y sus limitaciones

Existen distintos métodos disponibles para cuantificar la concentración de Lp(a), incluidos los inmunoensayos, la electroforesis y la espectrofotometría de masas. Sin embargo, estos métodos tienen limitaciones debido a la heterogeneidad de las partículas de Lp(a), que, como hemos comentado, pueden variar en tamaño y estructura [13], 15], 17]. Esto puede provocar diferencias en los resultados obtenidos por diferentes métodos, lo que dificulta la comparación de los datos de diferentes estudios.

Los métodos más ampliamente utilizados son los inmunoensayos: inmunoturbidimétricos y nefelométricos [43], 44]. Para que un método analítico sea adecuado, se ha de tener en cuenta lo siguiente:

– Número de repeticiones variables del KIV2 en la molécula de apo(a).
– Uso de anticuerpos policlonales que reconocen diferentes epítopos de la apo(a), que incluyen secuencias repetidas en un número variable, por lo que puede subestimarse o sobreestimarse la concentración de Lp(a) en función de la presencia de isoformas pequeñas o grandes de la lipoproteína.

Es difícil medir con precisión la concentración de Lp(a) debido al alto grado de variación de tamaño en la apo(a), causado por el número variable de repeticiones de KIV2. Resulta prácticamente imposible seleccionar calibradores de ensayo con el mismo tamaño de apo(a), presente en las muestras individuales a analizar. Esto puede provocar una sobreestimación de los valores de Lp(a) en muestras con moléculas de Lp(a) más grandes que las del calibrador y una subestimación de los valores de Lp(a) en muestras con moléculas de Lp(a) más pequeñas que las del calibrador [14], 43].

La masa de las partículas medidas no reflejará el número de partículas de Lp(a) debido a la variación de tamaño. Los calibradores de los ensayos turbidimétricos e inmunonefelométricos se seleccionan normalmente por tener concentraciones altas de Lp(a), pero las isoformas presentes en los calibradores pueden estar constituidas predominantemente por tamaños de apo(a) pequeños, lo que resulta en una sobreestimación de los valores de Lp(a) en la mayoría de las muestras. Además, no es posible determinar con precisión la masa total de la partícula heterogénea de Lp(a) porque requiere la cuantificación de todos los componentes independientes de Lp(a), incluidos la proteína, los múltiples lípidos y los componentes de carbohidratos [43], 45].

Los métodos que miden la concentración molar de Lp(a) miden la concentración de la partícula del componente principal que identifica la partícula de Lp(a), que es apo(a), sin el sesgo introducido por el tamaño de la partícula. En contraste, los ensayos de masa miden cantidades variables de todos los componentes de la masa de la Lp(a), incluyendo la sensibilidad a las diferencias en el tamaño de la isoforma de apo(a) [43].

Además, la proporción de estos componentes de la Lp(a) puede diferir entre los pacientes, lo que añade imprecisión a las mediciones. Por lo tanto, los ensayos de masa, que proporcionan los resultados en mg/dL, tienen una limitación inherente en la evaluación precisa de la concentración de partículas circulantes de la Lp(a) y pueden no ofrecer la suficiente calidad analítica en la medición desde un punto de vista clínico [43].

Para minimizar los problemas relacionados con la medición de la concentración de Lp(a), es importante seguir las pautas y recomendaciones de la Federación Internacional de Química Clínica y Medicina de Laboratorio (IFCC) y la Organización Mundial de la Salud (OMS) con el fin de garantizar resultados precisos y confiables [46].

El Grupo de Trabajo de la IFCC para Apolipoproteínas por Espectrometría de Masas (IFCC WG APO-MS) está trabajando en el desarrollo de métodos de medición para caracterizar con mayor precisión la apo(a) a nivel molecular. Este proceso incluye la creación de materiales de referencia primarios y secundarios para apolipoproteínas, incluída la Lp(a), utilizando un procedimiento de medición de referencia basado en cromatografía líquida-espectrometría de masas (LC-MS/MS). Este método permitirá definir inequívocamente la composición de la apo(a) y garantizará que los resultados de los procedimientos de medición comerciales de la Lp(a) o apo(a) sean verdaderamente trazables al Sistema Internacional [47].

La caracterización de un material de referencia secundario para ser utilizado por los fabricantes de los métodos disponibles comercialmente, permite asignar un valor objetivo de Lp(a), basado en la precisión, a sus calibradores. Dado que la determinación de la masa total de la Lp(a) es altamente heterogénea y compleja, se decide asignar un valor objetivo en nmol/L de proteína Lp(a), teniendo en cuenta el polimorfismo de tamaño de apo(a), que es el constituyente de la Lp(a) generalmente medido directamente por los inmunoensayos [48].

En el estudio de Dikaios et al. [49] sobre la estandarización de los materiales de referencia para la medición de la concentración de Lp(a), se evalúa la correlación entre el método de referencia candidato y los procedimientos de medida basados en inmunoensayos, así como la conmutabilidad de los diferentes materiales de referencia. Los resultados muestran la necesidad de estandarizar los procedimientos de medida de la concentración de Lp(a) para mejorar la atención a los pacientes.

En algunos estudios, se ha considerado como método de referencia para la medición de la concentración de Lp(a) el método desarrollado en *Northwest Lipid Metabolism and Diabetes Research Laboratories* (NLMDRL) de la Universidad de Washington [50], 51].

La medición de la concentración de Lp(a) mediante un ensayo estandarizado es el método de elección para estimar el riesgo asociado a esta lipoproteína aterogénica. Los ensayos que proporcionan la concentración de Lp(a) en mg/dL indican la masa de las partículas de Lp(a) de diferentes tamaños, mientras que los que indican nmol/L reflejan el número real de partículas. Dado que la unidad de medida más adecuada para la Lp(a) es la concentración en nmol/L, se recomienda no convertir los resultados entre mg/dL y nmol/L, ya que todos los factores de conversión dependen inherentemente de la isoforma de la Lp(a).

Para mejorar la armonización de los ensayos de medición de Lp(a), se debe:

– Verificar que la exactitud del ensayo haya sido certificada por el Laboratorio de Investigación de Lípidos del Noroeste en Seattle.
– No convertir los resultados de nmol/L a mg/dL.
– Utilizar programas de aseguramiento de calidad externos que distribuyan muestras con composición conocida de isoformas de apo(a) y valores de Lp(a) asignados por un método validado e independiente del polimorfismo de tamaño de apo(a) y calibración rastreable al material de referencia de la OMS/IFCC.
– Las muestras de control de calidad externo deben cubrir el intervalo de concentraciones clínicamente relevante de 90–200 nmol/L.

Actualmente, marcadores lipoproteicos avanzados como el número de partículas que contienen apoB100 han ganado importancia especialmente en la evaluación de riesgo de enfermedad cardiovascular residual. La técnica idónea para cuantificar el número de partículas, o concentración de partículas, es la resonancia magnética nuclear (RMN). Sin embargo, la RMN no puede distinguir las concentraciones de partículas de Lp(a) de las LDL [52], [53], [54].

Teniendo en cuenta todas estas cuestiones, resulta difícil realizar una comparabilidad entre diferentes metodologías analíticas. Un análisis reciente de ensayos con calibradores a cinco puntos mostró variaciones significativas entre laboratorios y entre ensayos, explicadas sólo parcialmente por el polimorfismo del tamaño de apo(a) [55].

La estrategia ideal para la cuantificación seria la obtención de un anticuerpo para un epítopo único no repetitivo en la apo(a), reconociendo cada partícula de Lp(a) una vez e informando los niveles como nmol/L. Marcovina et al. [56] han desarrollado un inmunoensayo que utiliza un anticuerpo monoclonal dirigido contra un único sitio antigénico, presente en el subtipo 9 de KIV.

**Figura 2:**
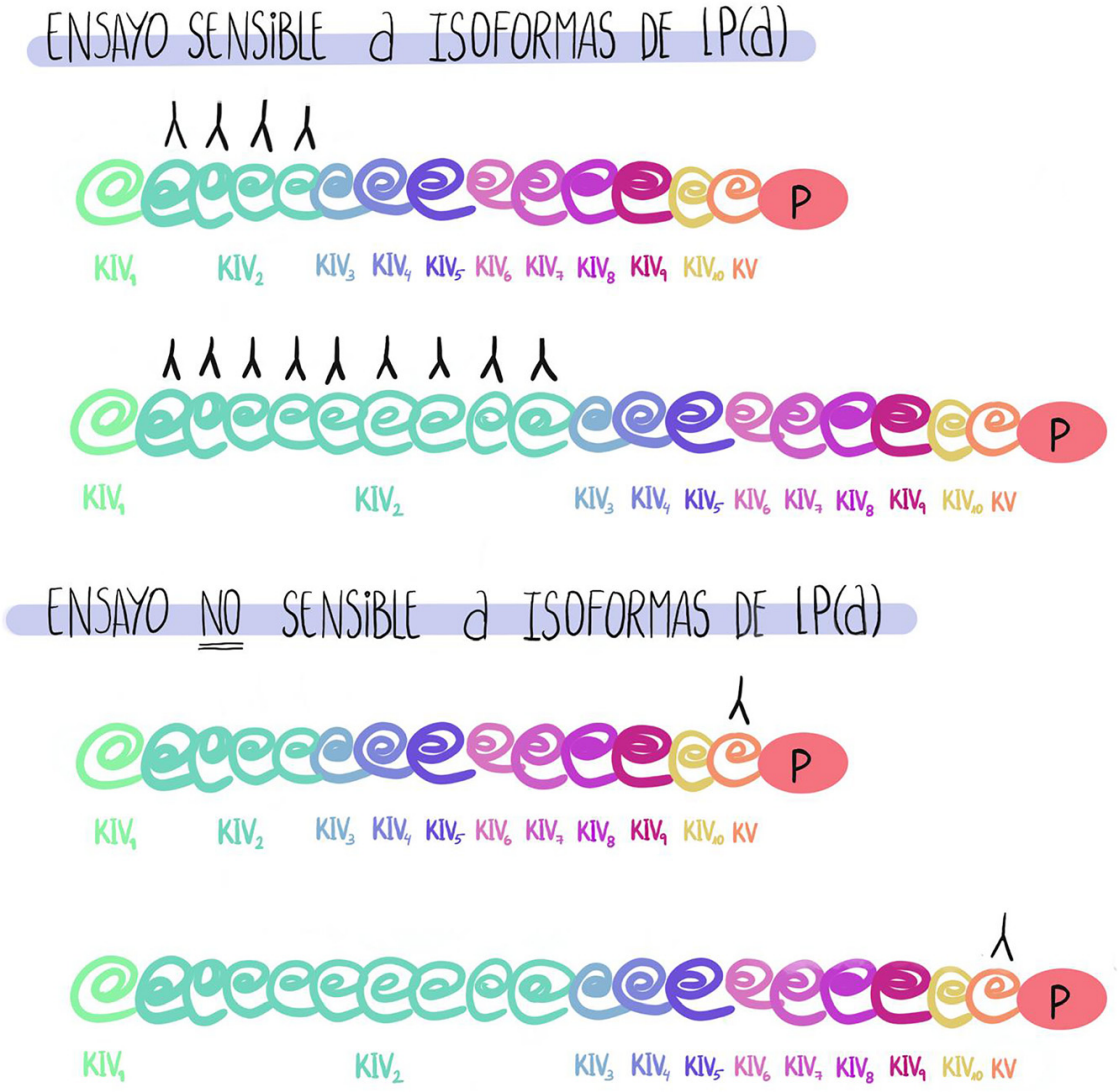
Representación esquemática de las diferencias metodológicas entre ensayos sensibles y no sensibles a diferentes isoformas de Lp(a).

En la Figura 2 se muestra gráficamente el uso de una metodología analítica sensible y otra no sensible a las diferentes isoformas de la Lp(a).

## ¿Se recomienda la corrección de LDL-C por Lp(a)?

Uno de los principales componentes de la partícula de Lp(a) es el colesterol el cual aproximadamente representa un 30–45 % del contenido de la partícula. En pacientes con concentraciones muy elevadas de Lp(a) este colesterol puede afectar a la estimación del LDL-C por los cálculos habituales, por lo que en ocasiones se ha utilizado el LDL-C corregido por Lp(a), habiéndose propuesto la modificación de Dahlen [57] de la fórmula de Friedewald con valores de Lp(a) determinados en mg/dL, y se ha utilizado en algunos estudios como el FOURIER [58]. Esta fórmula implica la medida de la Lp(a) en mg/dL o bien su conversión a estas unidades desde mmol/L.

En la práctica clínica actual, los métodos para determinar el LDL-C miden tanto el contenido de colesterol en las LDL como en la Lp(a). Existe un método avanzado, sensible y rápido que proporciona información sobre la relación masa/colesterol de la Lp(a), permitiendo una estimación más precisa del LDL-C y una reevaluación de su papel en la medicina clínica. El LDL-C se determina tras el aislamiento de la Lp(a) utilizando perlas magnéticas unidas al anticuerpo monoclonal LPA4, que reconoce la apolipoproteína(a). Este ensayo no detecta el colesterol en muestras de plasma sin Lp(a) y mantiene una linealidad hasta concentraciones de 747 nM de Lp(a).

Hay que tener en cuenta que, los sujetos con concentración de Lp(a) elevada tienen una menor respuesta a los fármacos hipolipemiantes como las estatinas, de hecho, una escasa respuesta hipolipemiante a estos fármacos es un motivo para sospechar un aumento de Lp(a) y solicitarla al laboratorio [59].

## ¿Qué papel debe jugar el laboratorio en el informe analítico?

Un detalle importante en el documento de la EAS (*European Atherosclerosis Society*) [26] es la relevancia que confiere al papel del laboratorio clínico en su procesamiento analítico y post-analitico, sugiriendo la inclusión en los informes analíticos de:

– Tipo de técnica analítica empleada, para permitir la correcta interpretación de los resultados discrepantes en el seguimiento del paciente.
– Instaurar alertas o comentarios interpretativos que aborden la implicación de la Lp(a) en la ECVA, sugerencia de solicitud de estudios en cascada familiar, y posible derivación a una unidad de lípidos.
– Valorar causas secundarias que puedan incrementar el valor de la Lp(a).

Ejemplos de comentarios interpretativos para la Lp(a):

– Lp(a)=120–180 mg/dL: Confiere un riesgo muy alto de infarto de miocardio y estenosis de la válvula aórtica [60], 61].
– Lp(a) >180 mg/dL: Confiere un riesgo de ECVA equivalente al riesgo asociado con la HFHe [60], 61].
– La Lp(a): Confiere un mayor riesgo de ECVA y no se modifica con los tratamientos hipolipemiantes habituales. Su monitorización no se recomienda, excepto en terapias dirigidas hacia PCSK9 [60], 61].
